# Genus *Calliandra*—*Calliandra portoricensis*, *Calliandra haematocephala*, *Calliandra surinamensis*: A Journey from Traditional Knowledge to Modern Experimental Studies in Disease Prevention and Treatment

**DOI:** 10.3390/ijms27041840

**Published:** 2026-02-14

**Authors:** Adedoyin O. Adefisan-Adeoye, Samson O. Kosemani, Olayinka A. Adebayo, Temitope D. Adeoye, Jeremiah O. Unuofin, Sogolo L. Lebelo, Oluwatosin A. Adaramoye

**Affiliations:** 1 Pediatric Brain Tumor Program, Department of Pediatric, Hematology-Oncology Section, Baylor College of Medicine, Houston, TX 77030, USA; 2 Chemical Sciences Department, Faculty of Computing and Applied Sciences, Dominion University Ibadan, Ibadan 200005, Nigeria; 3 Department of Immunology, Institute of Biomedical Science, University of Sao Paulo, São Paulo 05508-220, Brazil; kosemanisamson2014@gmail.com; 4 Department of Biochemistry, College of Applied and Natural Sciences, Mcpherson University, KM 96 Lagos-Ibadan Express Way, Abeokuta 110117, Nigeria; aoadebayor@gmail.com; 5 Federal Teaching Hospital, Ido-Ekiti 371101, Nigeria; temitopedennis@gmail.com; 6 Department of Life and Consumer Sciences, University of South Africa, Cnr. Christiaan de Wet and Pioneer Ave., Private Bag X6, Florida 1710, South Africa; unuofinjeremiah@gmail.com; 7 Molecular Drug Metabolism and Toxicology Laboratories, Department of Biochemistry, Faculty of Basic Medical Sciences, College of Medicine, University of Ibadan, Ibadan 200001, Nigeria; aoadaramoye@yahoo.com

**Keywords:** *Calliandra portoricensis*, *Calliandra haematocephala*, *Calliandra surinamensis*, cancer, antioxidants, anti-inflammation, oxidative stress, cardiovascular disease, antibacterial, antifungal, antimicrobial

## Abstract

The genus *Calliandra* (Leguminosae: Mimosoideae) encompasses over 200 species, many of which hold significant ethnobotanical value. However, a critical and comprehensive review consolidating their phytochemical and pharmacological knowledge is currently lacking. This article aims to provide a detailed and analytical overview of the traditional uses, phytochemistry, and pharmacological properties of the most studied *Calliandra* species, identifying trends, gaps, and future research priorities. A systematic literature search was conducted using Google Scholar, Scopus, Web of Science, and PubMed from 1986 to 2025. The review focuses on *Calliandra portoricensis*, *Calliandra haematocephala*, and *Calliandra surinamensis* due to the relative abundance of scientific literature concerning their medicinal applications. These species produce a diverse array of secondary metabolites, including distinctive galloylated flavonoids, phenolic acids, and triterpenes. Extracts and isolated compounds demonstrate a wide range of pharmacological activities, such as antioxidant, antimicrobial, anti-inflammatory, antidiabetic, and anticancer effects, providing a scientific basis for their traditional uses. The genus *Calliandra* represents a promising source of bioactive compounds. However, future research must focus on compound isolation, mechanistic studies, rigorous toxicological profiling, and clinical trials to fully realize its therapeutic potential.

## 1. General Introduction, Distribution, Taxonomy, and Utility

The genus *Calliandra* (Leguminosae: Mimosoideae) comprises over 200 species distributed across tropical regions, including the Americas, Africa, Madagascar, and India [1]. A significant taxonomic revision by Arias and colleagues categorized 132 species natives to the Americas into five subsections and fourteen series [2]. In horticulture, *Calliandra* is represented by 6–8 exotic species, which include several intraspecific taxa and natural hybrids. Wild species such as *Calliandra portoricensis* (*C. portoricensis*) (Jacq.) Benth., *Calliandra haematocephala* (*C. haematocephala*) (Hassk), and *Calliandra surinamensis* (*C. surinamensis*) (Willd) Benth. are notable in Africa. *Calliandra* genera are prized for their ornamental value, primarily due to their alternate leaves and distinctive spherical flowers characterized by elongated stamen [3]. In addition, *Calliandra* genera are believed to be protected by a phenomenon called nyctinasty, in which its leaves fold up at night [4]. Hummingbirds love the tree’s blossoms and use its nectar as their main food source. Because *Calliandra* grows well in dry environments, it is a great option for xeriscaping [5,6]. The tree serves as a host plant for the larvae of butterflies, hence sustaining their numbers. *Calliandra* is very simple to cultivate and once established, requires very little maintenance.

Despite their popularity as garden plants, only two species within this genus have been utilized in forestry applications. Among them, *Calliandra calothyrsus* (*C. calothyrsus*) (Meisn) stands out for its adaptability to large-scale plantations, attributed to its robust coppicing ability and nitrogen-fixing properties. Beyond ornamental and forestry uses, *Calliandra* genera contribute to diverse ecological and economic activities, offering products such as forage, honey, and fuelwood, while also serving roles in soil erosion control and soil fertility enhancement [7]. Morphological diversity within the genus has led to reports of hybrids and cultivars, such as those involving *C. haematocephala* and *Calliandra tweedii* (Benth), which exhibits varying color forms. Additionally, a hybrid between *C. calothyrsus* and *Calliandra houstoniana* (Mill.) Standl. was identified by Chamberlain and Hubert [6]. Given the prevalence of natural hybrids and morphological variation, molecular techniques are recommended to complement morphological evidence in the identification and classification of cultivated taxa. This approach ensures accurate delimitation and enhances understanding of the genus’ extensive diversity.

Recently, there has been growing research interest on underexplored medicinal plants as potential sources for discovering new secondary metabolites, especially those with huge therapeutic potentials. This new trend is driven partly due to drug-resistant and adverse reactions to chemotherapeutic agents, where traditional synthetic methods have produced a few novel drug candidates, and increased multidrug resistance has increased the public health burden. Consequently, research has increased investigations into phyto-active compounds such as terpenoids, alkaloids, phenolics to assess their possible pharmacological activities [3]. This development is encouraged by improvements in omics high-throughput screening and in silico molecular docking, which enables the identification and secondary metabolites from a phyto library. Based on extensive use and scientific evidence, the three most researched *Calliandra* species are *C. portoricensis*, *C. hematocephala*, and *C. sunarimensis*. This review will focus on these three *Calliandra* genera. It will conduct a thorough search for information on traditional uses, taxonomy, phytochemistry, and pharmacological uses, emphasizing their medicinal and pharmaceutical importance.

### 1.1. Methodology and Review Scope

**Figure 1:**
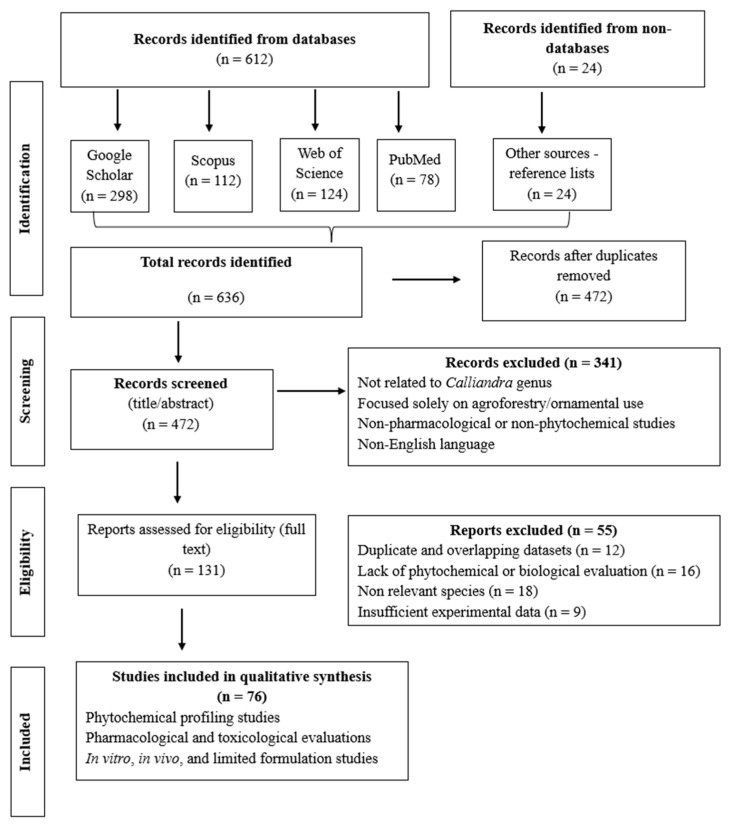
The PRISMA 2020 flow chart illustrates how studies on *C. portoricensis*, *C. haematocephala*, and *C. surinamensis* are identified, screened, eligible, and included in the systematic review of phytochemical and pharmacological investigations.

This review was compiled based on a systematic literature search conducted in online databases, including Google Scholar, Scopus, Web of Science, and PubMed. The search utilized keywords such as “*Calliandra*,” “*Calliandra* phytochemistry,” “*Calliandra* pharmacology,” and the specific species names, with no date restrictions applied initially, but focusing primarily on the literature from the last three decades. The PRISMA 2020 guidelines were adhered to while choosing the literature (Figure 1). Google Scholar, Scopus, Web of Science, and PubMed were used to find records. Following the elimination of duplicates, full-text eligibility was evaluated using predetermined inclusion and exclusion criteria after titles and abstracts were examined. The results were synthesized qualitatively, and no meta-analysis was carried out due to the variability in experimental designs, outcomes, and models.

While the genus is large, this review deliberately focuses on *C. portoricensis*, *C. haematocephala*, and *C. surinamensis*. This focused scope is justified as these three species have been the subject of the most intensive phytochemical and pharmacological investigations related to human health, forming a substantial and analyzable body of literature. Other species, such as *C. calothyrsus*, are more widely studied in an agroforestry context. This approach allows for a deeper, more critical analysis of the best-characterized medicinal species rather than a superficial overview of the entire genus. The inclusion criteria were peer-reviewed articles in English, while unpublished theses and non-English papers were excluded.

### 1.2. Traditional Uses of Calliandra Genera

Traditional uses of *C. portoricensis* (Jacq.), a sprawling perennial shrub belonging to the Mimosaceae family, are notable in ethnomedicine. Most of this shrub is found in the West Indians, Panama, Africa, and Mexico [6,8]. It is also present in several tropical countries, including Nigeria, Ghana, and Côte d’Ivoire. According to Akah and Nwaiwu [9] and Enwuru et al. [10], this herb has been a staple in traditional Nigerian medicine for many years. Practitioners of traditional herbal medicine use the plant as a laxative and worm expeller to treat convulsions, lumbago, constipation, gonorrhea, and more [9]. Agunu et al. [11] indicate that it possesses anticonvulsant, analgesic, antidiarrheal, antispasmodic, and antipyretic properties. *C. portoricensis* has received scientific validation for a range of traditional applications, including antibacterial, antiproliferative, antiulcer, and chemopreventive functions [12,13,14]. Traditional medicine practices in Nigeria extensively use *C. portoricensis* extracts to address diverse ailments. For instance, it treats breast engorgement, toothaches, coated tongues, swollen tonsils, tuberculosis, convulsions, diarrhea, infections, and sickle cell crises [15,16,17,18]. Besides its well-known white flowers, this plant has been utilized in traditional medicine for its anti-inflammatory, antibacterial, and antifungal properties [19,20]. Substantial findings highlighted its efficacy against fever, breast engorgement, gastrointestinal disorders, amenorrhea, and lumbago [21,22,23,24,25]. In Ghana, the root bark of *C. portoricensis* is combined with pepper for treating gonorrhea, alleviating headaches, and preparing ophthalmic remedies.

The medicinal properties of *C. portoricensis* encompass its ability to reduce fever and alleviate pain, as well as its efficacy in treating wounds, skin conditions, and respiratory issues [9,11,19,20]. Traditionally, *C. portoricensis* can be utilized to enhance general health and well-being through herbal preparations such as teas, tinctures, and infusions [22,23,24]. In addition to its numerous other benefits, *C. portoricensis* can assist with blood sugar regulation, alleviate symptoms of anxiety and depression, address digestive issues, and provide relief for snake bites [26]. Historically, powdered roots or alcoholic extracts of *C. portoricensis* leaves have been utilized for snake bite treatments [26]. Over time, traditional herbalists in southeastern Nigeria developed methods for effectively managing carpet viper venoms. This particular recipe, an ethanolic extract of *C. portoricensis*, is specifically intended for treating snakebite hemotoxins that cause hemotoxicity (e.g., the hemotoxins of true vipers like the puff adder and the carpet viper, as well as pit vipers like rattlesnakes). According to traditional herbalists, the secondary metabolites (bioactive principles) of *C. portoricensis* do not affect the neurotoxins from elapine snakes, such as cobras, mambas, and coral snakes [26]. Regular consumption of *C. portoricensis* has also been linked to improved cardiovascular health, reduced risk of various cancers, and enhanced cognitive function [9,16,17,27]. By incorporating this plant into daily routines, individuals can experience better sleep, increased vitality, and an overall higher quality of life. The antioxidant properties of *C. portoricensis* can help prevent cellular damage, potentially lowering the risk of chronic diseases like cancer and heart disease [28,29]. This also allows individuals more time to engage in their hobbies and spend time with loved ones. Furthermore, its traditional use as an antifungal and antiseptic agent has been reported to prevent infections, enabling individuals to maintain their overall health and dental wellness while feeling more confident and comfortable in their daily lives [12,30].

Traditionally, *C. haematocephala* has been used mostly for ornamental and cultural purposes. Because of its vivid red, brush-like blossoms, which are thought to be symbols of beauty, vigor, and hospitality, the plant is frequently cultivated in home gardens, institutional landscapes, and public areas. Flowering branches are utilized as decorations at social events, ceremonies, and celebrations in certain local customs [31,32]. Because it is thought of as a plant that continuously adds color and vitality to its surroundings, the plant’s lengthy flowering period further increases its cultural importance. In addition, *C. haematocephala* is employed as an antibacterial agent in ethnomedicine [30]. Its floral extracts demonstrate both antibacterial and anti-helminthic properties [33,34,35]. *C. haematocephala* has been utilized in localized folk medicine in addition to its aesthetic value, though these applications are few and mostly empirical. Leaf infusions or decoctions have long been used in some tribes to improve overall health or ease minor digestive issues. Crushed leaves or bark formulations have occasionally been administered externally to small wounds, insect bites, or skin irritations, reflecting common ethnobotanical practices that use easily accessible plants for straightforward cures [30,33,36,37]. These applications lack substantial scientific validation and are frequently impacted by knowledge of related *Calliandra* species. The phenolics, flavonoids, and tannins found in *C. haematocephala* leaf extract provide significant antibacterial activity against various bacterial strains [30,36]. The decoction of the flower extract is used as a tonic and blood purifier traditionally due to its antioxidant properties [38]. Its roots can be used to treat hemorrhoids [39]. The *C. haematocephala* effervescent granules have been formulated for treating stomach ulcers [40,41].

The pigment extracted from *C. surinamensis* flowers is used as a dye in the manufacture of certain pharmaceutically significant medications [3]. *C. surinamensis* is also widely recognized in ethnomedicine for its decorative and horticultural applications in the tropics. The large, multi-trunked shrub *C. surinamensis* possesses several distinctive characteristics [42]. Furthermore, *C. surinamensis* has been used traditionally as an aqueous infusion to treat fever and overall body weakness [43]. According to certain ethnomedical sources, it may have anti-inflammatory and calming effects on the respiratory tract when used to treat respiratory symptoms including cough and mild bronchial infections [44]. Specifically, stem bark is used in traditional medicine to treat various ailments and diseases, including inflammation, infections, coughs, and wounds [44]. In addition to its therapeutic uses, the plant has cultural value as an ornamental species and is occasionally included into agroforestry systems, where its capacity to fix nitrogen enhances soil fertility. Compared to other *Calliandra* species, there is still little scientific evidence to support the wide range of therapeutic applications suggested by traditional knowledge [45].

Generally, the genus *Calliandra* enjoys global prominence, albeit its limited use in Central America has spurred much speculation. Historically, *Calliandra* trees have played a vital role in the environment, providing unique benefits that make them invaluable members of any ecosystem. *Calliandra* genera engage in a symbiotic relationship with nitrogen-fixing bacteria in their roots. Consequently, they require less chemical fertilizer as they enhance soil fertility. Moreover, the colorful flowers of the tree attract pollinators such as bees, butterflies, and hummingbirds, encouraging biodiversity. Due to its ability to serve as a windbreak, improve soil quality, and provide shade, *Calliandra* is frequently employed in agroforestry systems. Its fast growth and high calorific value also make it a preferred source of firewood in many rural communities.

### 1.3. Bioactive Phytochemicals Present in C. portoricensis, C. hematocephela and C. surinamensis

The roots, leaves, and stems of the *Calliandra genera* are the main parts used traditionally. These parts are rich in fatty acids, phytochemicals such as saponins, flavonoids, and other active compounds, and they have a pungent fragrance, a hint of bitterness, and warming qualities [18,26]. Different parts of the *Calliandra genera* contain different kinds of important chemicals, making it a rich source of these compounds. A total of about 70 compounds in all have been found in different parts of *C. portoricensis*, *C. haematocephala*, and *C. surinamensis* by the use of Gas Chromatography–Mass Spectrometry (GC-MS) (Table 1). According to Adefisan et al. [17,27], GC-MS investigations showed that the primary ingredients in the essential compounds of *C. portoricensis* contained majorly 22 compounds in the methanol and chloroform extracts of *C. portoricensis*. Methyl-3-phenylindole, Methyl stearate, Methyl 9-cis, 11-trans-octadecadienoate, Cis-13-Octadecenoic acid, D-Fructose, 3-*O*-methyl-1-Octadecene, Hexadecanoic acid, methyl ester, D-Fructose, 3-0-methyl-, Thiophene, 2-ethyltetrahydro-, B-d-Mannofuranoside, methyl tetraacetate, 1-Butene, 1-(methylthio)-, Trimethylsilyl 23 acetoxy-3,6,9,12,15,18,21-heptaoxatricisan-1-oate and Methyl tetra decanoate are among the twelve major compounds identified in the *C. portoricensis* methanol extracts (Table 1). Adaramoye et al. [16] and Oyebode et al. [13] equally reported 10 compounds in the methanol roots and leaf extracts of *C. portoricensis* using GC-MS characterization. Orishadipe and associates [12] analyzed the hexane extract of *C. portoricensis* using GC-MS and discovered that 14-methyl methylpentadecanoate was the primary constituent (Table 1). They also reported trace quantities of fatty acids and fatty acid methyl esters, including hexadecanoic acid, methylhexadecanooate, 9-oxo-methyl nonanoate, and a few more.

Numerous analyses of *C. haematocephala*’s chemical components have been conducted. The leaves and stem of *C. haematocephala* were found to contain three acylated quercetin rhamnosides, whose structures were determined to be quercitrin 2′-*O*-caffeate, quercitrin 3′-*O*-gallate, and quercitrin 2′,3′-di-*O*-gallate [48] (Table 1). Also, 17 known compounds were reported from *C. haematocephala* stem extract by Mohorram et al. [48]; they are gallic acid, methyl gallate, myricitrin, quercitrin, myricetin 3-*O*-â-D-4C1-glucopyranoside, isoquercitrin, myricetin afzelin, 3-*O*-(6′-*O*-galloyl)-â-D glucopyranoside, myricitrin 2′-*O*-gallate, quercitrin 2′-*O* gallate, afzelin 2′-*O*-gallate, myricitrin 3′-*O*-gallate, afzelin 3′-*O*-gallate, 1,2,3,4,6-penta-*O*-galloyl-â-D 4C1-glucopyranose, myricitrin 2′,3′-di-*O*-gallate, quercetin 3-*O*-methyl ether [48]. In addition, Nia et al. [35] documented the identification of betulinic acid and caffeineic acid from *C. haematocephala* (Table 1). Overall, limited research has been done on the significant compounds of *Calliandra genera* based on their separation from various plant sections. Nonetheless, their pharmacological effects and other biological properties have been widely investigated.

## 2. *Calliandra portoricensis*

### 2.1. Morphological Description and Distribution

**Figure 2:**
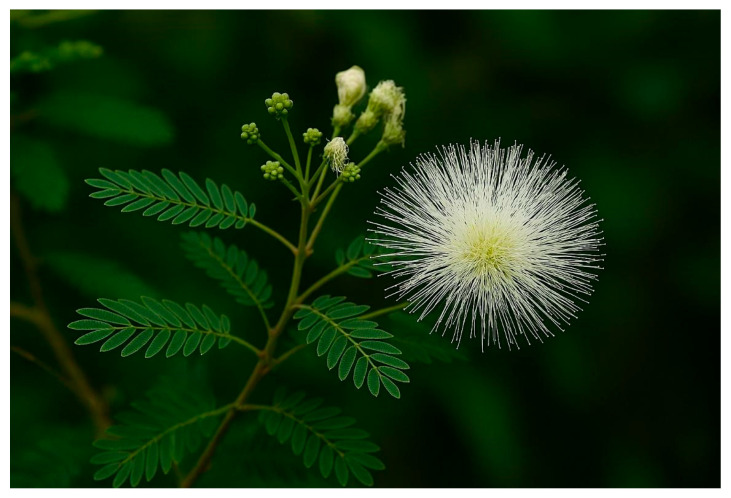
Botanical representation of *Calliandra portoricensis*.

*C. portoricensis*, commonly known as powder puff, is a perennial shrub belonging to the Leguminosae family. It is well-known for its fragrant, snowball-shaped white blossoms and has many medicinal uses (Figure 2). In Nigeria, the plant is referred to as “Tude” in Yoruba, “Ule” in Igbo, and “Oga” in Hausa. Globally, the genus *Calliandra* encompasses over 200 species distributed across tropical and subtropical regions of the Americas, Asia, and Africa, with notable examples including *C. eriophylla*, *C. anomala*, *C. haematocephala*, and *C. portoricensis* [50]. *C. portoricensis* is native to Mexico, Panama, and the West Indies. It produces delicate, fluffy white flowers in the spring, which are characterized by small, inconspicuous petals and white stamens that give the flower head its fluffy snowball appearance. In cooler climates, it can be grown in glasshouses. It can grow in most soil types if they are well-drained, and it only needs occasional watering in the winter. It requires minimal pruning to maintain its shape and remove dead branches. It is vulnerable to mealy bugs and red spider mites. Despite its broad distribution and ease of cultivation, the botanical and ecological literature on *C. portoricensis* remains largely descriptive, with limited integration of morphological variation, ecological interactions, and phytochemical diversity. This lack of critical comparative studies makes it challenging to relate its morphological features to its medicinal potential or environmental adaptability. Future research should adopt integrative approaches via combining molecular taxonomy, ecological assessment, and chemical profiling to clarify variations and their implications for pharmacological research.

### 2.2. Phytochemistry of Calliandra portoricensis

The phytochemical composition of *C. portoricensis* has been extensively analyzed, revealing the presence of several bioactive compounds (Table 1), including alkaloids, saponins, tannins, flavonoids, polyphenols, glycosides, phlobatannins, and reducing agents [51]. However, studies indicate the absence of tannins, phlobatannins, anthraquinones, and hydroxymethyl anthraquinones in both fresh and dried *C. portoricensis* extracts [9]. Notably, alkaloids and glycosides are consistently identified in *C. portoricensis* extracts, though the role of alkaloids, often regarded as highly toxic, remains debatable regarding their significance in plant metabolism [51]. While the presence of flavonoids, steroids, polyphenols, saponins, and reducing compounds has been repeatedly confirmed (Table 1) [28,52], most studies rely on preliminary qualitative screening or GC–MS analysis, with limited follow-up through bioguided assay fractionation or structure-activity relationship studies. This limits the ability to directly correlate specific compounds with observed biological activities. A more critical and standardized approach to phytochemical characterization, integrating advanced analytical techniques and quantitative assessments, is therefore needed to fully understand the chemical basis of the plant’s ethnomedicinal uses and therapeutic potential.

### 2.3. Biological Activities of Calliandra portoricensis

#### 2.3.1. Antimicrobial and Anti-Ulcer Activities

Several in vitro studies have reported the antimicrobial properties of *C. portoricensis*, Aguwa and Lawal [53] were the first to report that both ethanol and aqueous extract of the leaves is highly active against bacterial pathogens. Subsequently Oguegbulu et al. [54] comparatively studied five medicinal plants, including *C. portoricensis*, and result showed that *C. portoricensis* demonstrated the widest spectrum of activity, with ethyl acetate fractions showing the most potent inhibition. The zones of inhibition ranged from 14 to 22 mm, and minimum inhibitory concentrations (MICs) dropped between 25 and 100 µg/mL against *Staphylococcus aureus*, *Escherichia coli*, *Klebsiella pneumoniae*, and *Candida albicans*, surpassing ciprofloxacin and fluconazole controls. A complementary study by Orishadipe et al. [12] reported that the n-hexane root extract of the leaves inhibited *S. aureus* and *E. coli* with MICs of 100–200 µg/mL, though activity was absent against *Pseudomonas aeruginosa* and *Bacillus subtilis*. In vitro micro-dilution assay of peptide-rich and crude methanol root extracts found MIC in the low μg/mL range (e.g., MICs 12.25–50 μg/mL against MRSA and *S. aureus*; 25 μg/mL against *E. coli* in the methanol crude and peptide fractions [8]. Early pharmacological study on *C. portoricensis* showed that it also possesses anti-ulcer and gastro-protective activity. Intraperitoneal administration of ethanolic and aqueous leaf extracts reduced ulceration in pylorus-ligation and stress (cold-restraint) models at 50 mg/kg intraperitoneal, and the reported intraperitoneal LD_50_ values were 120.2 mg/kg (ethanolic) and 79.4 mg/kg (aqueous) [53]. Overall, it can be confirmed from these findings that *C. portoricensis* is a promising antimicrobial agent.

#### 2.3.2. Anticonvulsant and Analgesic Activities

In traditional medicine, *C. portoricensis* has been employed for managing stomach disorders and convulsions. Akah and Nwaiwu [9] were among the first to document its anticonvulsant properties. In their study using pentylenetetrazole and maximal electroshock seizure models, they demonstrated that aqueous extracts from the root and stem displayed protection upon administration to experimental animals. Also, they found out that alkaloid fraction produced no anticonvulsant effect up to 316 mg/kg intraperitoneal, indicating the active anticonvulsant constituents are non-alkaloidal. Their study was further validated by [15,29]. The analgesic potential of *C. portoricensis* was evaluated by Agunu et al. [11] using acetic acid-induced writhing and formalin-induced pain tests in rodents. Both roots and leaves of *C. portoricensis* showed significant analgesic effects (when given orally at 200, 400 and 600 mg/kg), reducing abdominal cramps and formalin-provoked pain, highlighting its therapeutic promise for pain management.

#### 2.3.3. Antioxidant and Anti-Venom Properties

The antioxidant and anti-venom properties of *C. portoricensis* have also drawn significant attention [14,16,17,26,27,55,56]. Ethanol extracts of *C. portoricensis* have long been used by herbalists in Nigeria for snakebite management. Onyeama et al. [26] evaluated *Wistar* rats challenged with Echis ocellatus venom (0.2 mg/rat, intramuscularly) and subsequently treated with flavonoid, polyphenol fractions, or whole ethanolic extract of *C. portoricensis* (100 mg/100 g body weight, intramuscular) revealed that *C. portoricensis* extracts mitigated oxidative stress caused by venom in rats by reducing phospholipase A_2_ activity from 0.2104 μmol/min/mL in venom control to 0.1195, 0.0779, and 0.0536 μmol/min/mL in flavonoid, polyphenol, and whole extracts, respectively. It also normalized liver enzyme activities (amino transferases), improved haematological indices (hemoglobin, red blood cell, white blood cell, platelets), and enhanced endogenous antioxidant defenses (superoxide dismutase, glutathione peroxidase). Co-administration of *C. portoricensis* was shown to reverse venom-induced hepatotoxicity and improve hemoglobin levels. Adaramoye et al. [16] also confirmed the antioxidative potential of *C. portoricensis*’s methanol extract in vitro, supporting its therapeutic use for oxidative stress and venom-related conditions. In addition, Adefisan and colleagues extensively documented the antioxidative activities of *C. portoricensis* in chemical-induced breast cancer and reproductive toxicities [14,17,27,55]. They demonstrated that the methanol root bark and chloroform fraction of *C. portoricensis* substantially improved antioxidant activities in mammary tumor-bearing rats.

#### 2.3.4. Anti-Proliferative and Cytotoxic Effects

The anti-proliferative properties of *C. portoricensis* against cancer cells were demonstrated by Adaramoye et al. [16], who treated PC-3 and LNCaP prostate cancer cells with *C. portoricensis* root methanol extract at 10, 50 and 100 μg/mL. Growth inhibition (48 h) was dose-dependent: PC-3: 7% (10 μg/mL), 74% (50 μg/mL) and 92% (100 μg/mL); LNCaP: 27% (10 μg/mL), 73% (50 μg/mL) and 85% (100 μg/mL). The extract showed low toxicity at its half-inhibitory concentration and 50 μg/mL produced significant anti-angiogenic effects in the chorioallantoic membrane assay [16]. Additionally, Oyebode et al. [13] found that methanol extracts of *C. portoricensis* effectively inhibited the proliferation of prostate cancer cells (LNCaP and DU-145) while sparing normal VERO cells, underscoring their potential as a selective cytotoxic agent. Moreover, Adefisan et al. [27,55] used MCF-7 cells to show the anti-proliferative properties of the chloroform fraction of *C. portoricensis* both in vitro and in vivo. According to their findings, *C. portoricensis* causes MCF-7 cells to undergo apoptosis and alters the *N*-methyl-*N*-nitrosourea and benzo(a)pyrene carcinogenesis processes in rats [27,55]. Similarly, Kosemani et al. [57] and colleagues’ research also showed that the methanol fraction of *C. portoricensis* uses anti-inflammatory and anti-oxidative pathways to protect the mammary gland from 7,12-dimethyl-benz[a]anthracence assaults. All these findings point to *C. portoricensis*’ antiproliferative potential.

#### 2.3.5. Safety and Toxicity of *Calliandra portoricensis*

The long-term administration of *C. portoricensis* root and leaf extracts has been reported to affect gastrointestinal and pancreatic functions in animal models, specifically mice and rats [4]. Acute toxicity studies by Onyeama et al. [26] examined the lethal dosage of *C. portoricensis* extracts. Their findings indicated significant mortality rates at administered doses of 625 mg/kg and 10,000 mg/kg. At a lower dose of 39 mg/kg, only one death was observed within 24 h, highlighting the dose-dependent toxicity of *C. portoricensis*. However, most toxicity studies reported are limited to acute and sub-acute animal experiments, with little emphasis on mechanistic insights, chronic exposure effects, or organ-specific toxicity investigations. Biomarkers like haematological alterations, histopathological changes, and biochemical indices have not been comprehensively assessed. Furthermore, standardized toxicity protocols and comparisons across different plant parts or extraction solvents are lacking, making it difficult to establish safe therapeutic margins. Rigorous toxicological profiling, including chronic toxicity, genotoxicity, and pharmacokinetic studies are essential to support the safe development of *C. portoricensis*-based therapeutic applications.

## 3. *Calliandra haematocephala*

### 3.1. Morphological Description and Distribution

**Figure 3:**
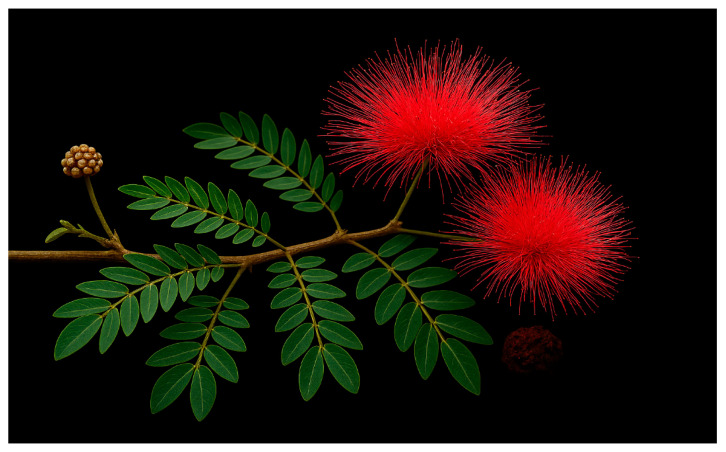
Morphological features of the leaves and flowers of *Calliandra haematocephala*.

*Calliandra haematocephala* (Haskk), commonly known as the powder puff tree, is an ornamental shrub or small tree reaching heights of 1–3 m from the family, Fabacea, and subfamily of Mimosoideae (Figure 3). Its glossy, pinnately compound green leaflets, with five to ten pairs, are oblong and acute [42]. The branches are brown, cylindrical, and textured. Notable for its striking flowers, the plant produces puff-like inflorescences in shades of watermelon pink, deep red, or white during warm months (Figure 3). Each flower cluster, measuring up to 7 cm in diameter, contains numerous long, silky stamens that transition from silky to dark metallic green over time. The fruit is a linear-oblanceolate pod, 6–11 cm long and 5–13 mm wide, that splits upon ripening to release oblong brown seeds [33,39]. It is indigenous to tropical America and India and can be planted in parks and gardens. While its morphological features are well-documented, the taxonomic and ecological studies on *C. haematocephala* remain limited, particularly on variations and adaptive traits. There is also a lack of research linking these morphological characteristics to the plant’s chemical or pharmacological properties, despite increasing interest in its medicinal potential. Comparative analyses with other *Calliandra* species could clarify how structural variations influence ecological interactions and secondary metabolite production. Integrating morphological, molecular, and phytochemical data would provide a more comprehensive understanding of this species beyond its ornamental value.

### 3.2. Phytochemical Constituents Calliandra haematocephala

Comprehensive studies reveal a diverse phytochemical profile in *C. haematocephala* (Table 1). Numerous phytochemical investigations of *C. haematocephala* have been conducted in recent decades, and the results have indicated that flavonoids, carbohydrates, alkaloids, glycosides, saponins, steroids, and tannins are among the main categories of substances found in the plant [42]. Extracts from different parts of the plant contain phenolics, flavonoids, alkaloids, saponins, tannins, glycosides, and steroids. Specific isolated compounds include lupeol, betulinic acid, and flavonoids such as quercetin, kaempferol, and myricetin. The plant’s tannins, predominantly procyanidins and prodelphinidins, show significant antioxidant potential. In addition, volatile compounds make up 93.32% of the plant’s total aromatic profile, with oxygenated constituents contributing to its biological activities [34]. Studies have identified various phytochemicals in *C. haematocephala*, including flavonoids, tannins, terpenoids, glycosides, and volatiles. Flavonoid glycosides such as quercetin-3-*O*-rhamnopyranoside, kaempferol-3-*O*-(2″-*O*-galloyl)-rhamnopyranoside, and myricetin-3-*O*-(2″,3″-di-*O*-galloyl)-rhamnopyranoside have been reported (Table 1) [37,49,58,59]. The plant’s phytochemical profile contributes to its significant biological activities.

### 3.3. Biological Activities of Calliandra haematocephala

#### 3.3.1. Phytochemical Constituents, Cytoprotective, Antioxidant and Antimicrobial Potential of *C. haematocephala*

The bioactive profile of *C. haematocephala* has attracted significant scientific attention due to its diverse pharmacological activities, including antioxidants, antimicrobial, antibacterial, antifungal, anti-inflammatory, and cytoprotective properties [30,33,34,36,37]. Research has shown that both crude extracts and isolated compounds contribute to their broad therapeutic value across multiple biological systems. Moharram et al. [48] and Gupta et al. [37] identified three new acylated quercetin rhamnosides, quercitrin 2-*O*-caffeate, quercitrin 3-*O*-gallate, and quercitrin 2″,3″-di-*O*-gallate along with 17 known compounds. These flavonoids exhibited moderate to strong radical scavenging properties, while myricitrin and quercitrin demonstrated cytotoxic effects on Artemia salina, supporting the antioxidant and cytoprotective potential of the species. Similarly, Antony de Paula Barbosa [60] demonstrated the gastroprotective effect of butanolic extract in gastric lesions induced by acidified alcohol, where the extract reduced hemorrhage and necrosis, likely due to its cytoprotective constituents. Collectively, these studies confirm that *C. haematocephala* possesses potent antioxidant and cytoprotective properties, although variations in extraction methods and solvent polarity may account for differences in reported activity levels. Furthermore, antibacterial investigations by Punnagai et al. [33] revealed inhibition against *Salmonella typhi* (12 mm), *Serratia marcescens* (9 mm), and *Staphylococcus aureus* (9 mm), comparable to ampicillin, findings that align with those of Srishti et al. [61]. The consistency of these results underscores the broad-spectrum antimicrobial potential of the plant, though further work is needed to isolate and characterize its most active constituents.

#### 3.3.2. Antiviral, Antihelmintic, and Hepatoprotective Activities

Beyond its antioxidant effects, *C. haematocephala* has demonstrated remarkable antiviral, antiparasitic, and hepatoprotective properties [58,62]. In a recent study, Shaheen et al. [58] reported that methanol extracts of *C. haematocephala* leaves significantly reduced mortality and diarrhea severity in rotavirus-infected mice, highlighting its potential antiviral efficacy. In parallel, Jharna Tiwari and Gopal Rai [63] observed strong anthelmintic effects from flower extracts tested against Eisenia foetida; these findings were later corroborated by Ajay Kumar Shukla [64] using Albendazole as a control. Together, these studies reveal the plant’s broad bioactivity against viral and parasitic agents, though its precise mechanism of action remains poorly defined. Additionally, Abo-Elhamd et al. [62] documented the hepatoprotective influence of total alcoholic extract against carbon tetrachloride-induced liver injury, demonstrating reductions in alanine aminotransferase, aspartate aminotransferase, and bilirubin levels. These results support the idea that the polyphenolic and flavonoid-rich composition of *C. haematocephala* underlies both hepatic and systemic protection, complementing its antioxidant profile.

#### 3.3.3. Wound-Healing, Antisickling, and Antidiabetic Potentials

Emerging research has also revealed the regenerative and metabolic benefits of *C. haematocephala.* Gupta et al. [37] found that ethyl acetate extract of *C. haematocephala* leaves enhanced wound healing by increasing tensile strength, hydroxyproline, and protein levels in healed tissues. This observation is consistent with Abo-Elhamd et al. [62], who also attributed tissue protection to high flavonoid content. Such consistency suggests that the plant’s secondary metabolites are multifunctional, having antioxidant, anti-inflammatory, and wound-healing benefits. Amujoyegba et al. [15] evaluated the antisickling properties of *C. haematocephala* and *C. portoricensis*, finding that ethanolic root extract exhibited the highest inhibitory (90.19%) and reversal (92.63%) effects on sickled human red blood cells. The results indicate that the bioactive compounds in *C. haematocephala* possess potential for hemoglobin reconfiguration, aligning with its previously reported antioxidant activity, as oxidative stress is central to red blood cell sickling. Punnagai and Glory Josephine [39] demonstrated the alpha-amylase and alpha-glucosidase inhibitory potential of *C. haematocephala* leaves, suggesting its role in diabetes management. This aligns with evidence of its polyphenolic richness, as phenolic compounds are known to modulate carbohydrate metabolism. However, unlike the studies of Moharram et al. [48], which focused on quercetin derivatives, Punnagai and Glory Josephine [39] did not isolate specific constituents, leaving a gap in understanding the exact bioactive principles responsible for the antidiabetic effect.

#### 3.3.4. Nanotechnological Applications and Anti-Inflammatory Properties

The versatility of *C. haematocephala* extends into nanotechnology and inflammation modulation. Raja et al. [36] synthesized silver nanoparticles from *C. haematocephala* leaf extract, which exhibited antibacterial activity against *Escherichia coli* and potential applications in biosensors. Similarly, Ramesh et al. (2020) reported the synthesis of zinc oxide nanoflowers using *C. haematocephala* extract, showing excellent photocatalytic dye degradation properties [65]. These studies highlight the growing use of *C. haematocephala* in nanotechnology-based biomedical applications, though biocompatibility and toxicity profiles remain underexplored. Abou Zeid et al. [46] demonstrated that ethanol extract of *C. haematocephala* aerial parts exhibited 87.80% anti-inflammatory potency, attributed to its high quercetin content. Quercetin inhibits cyclooxygenase and lipoxygenase, reducing prostaglandin and leukotriene production [46]. This mechanistic explanation supports earlier reports by Moharram et al. [48] on the anti-inflammatory contribution of flavonoids. Nevertheless, variability in experimental models and dosing regimens across studies complicates direct comparison of efficacy levels. Srishti et al. [61] showed that methanolic extracts of *C. haematocephala* exhibited antibacterial effects against acne-*causing S. aureus* and *S. epidermidis*, comparable to benzoyl peroxide and clindamycin. This finding strengthens the antibacterial claims of Punnagai et al. [33], but both studies lacked detailed phytochemical quantification, which limits reproducibility.

#### 3.3.5. Pharmaceutical Formulation Potential and Future Research

Given its rich bioactive profile, *C. haematocephala* has also been explored for its pharmaceutical formulation potential, Gupta et al. [37] formulated herbal effervescent granules using *C. haematocephala* leaf extract, which demonstrated excellent flow properties, including good angle of repose, Carr’s index, and bulk density. The formulation potential of the extract further supports its pharmaceutical value. Collectively, while most studies affirm the pharmacological and technological versatility of *C. haematocephala*, comparative analysis reveals that methodological variations, particularly in solvent systems, assay types, and extract concentrations contribute to differences in reported efficacy. Therefore, future investigations should aim for standardized extraction and bioassay protocols to validate these promising results and facilitate clinical translation.

## 4. *Calliandra surinamensis*

### 4.1. Morphological Description and Distribution

**Figure 4:**
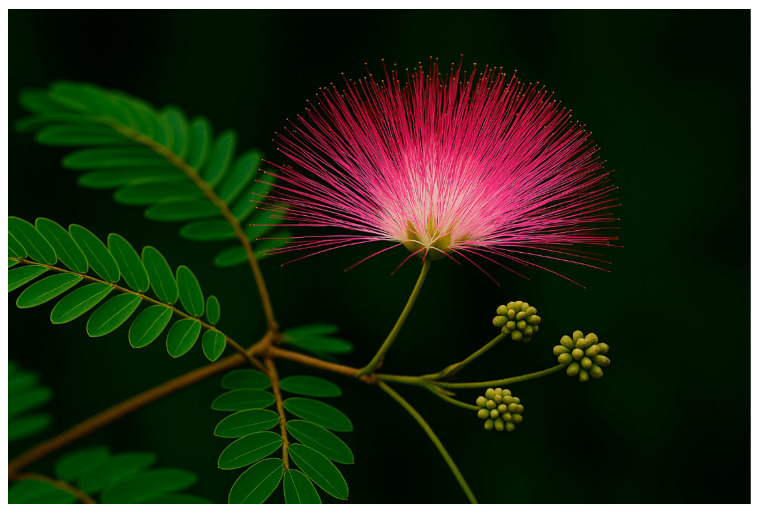
Leaf and floral morphology of *Calliandra surinamensis*.

*Calliandra surinamensis* (Wall.), a member of the Fabaceae family, is a flowering plant of significant ecological and economic importance. This evergreen, low-branching tropical shrub, commonly known as pink powder puff or Suriname powder puff, derives its name from Suriname, a country in Northern South America. Locally, it is referred to as “Kaliandra bunga merah” in Malaysia. The species is endemic to various regions, including southern Asia, Africa, Australia, and the Americas [66]. It may grow up to 5 m tall, have lovely evergreen foliage, and produces stunning pink blooms in a “powderpuff” spherical shape (Figure 4). The plant is renowned for its year-round floral display and is occasionally used as a hedge. *C. surinamensis*, commonly known for its use in traditional medicine, has a long history of application in treating various ailments due to its reported antioxidant and antimicrobial properties. The stem bark of *C. surinamensis* has been especially notable for its therapeutic potential [44]. Despite its prominence in ethnomedicine, research focusing on the chemical constituents of the plant remains limited. Preliminary studies conducted by Falodun and colleagues have identified several secondary metabolites in the root bark, including flavonoids, β-sitosterol 3-*O*-β-D-glucopyranoside, and recursterol, pointing to its significant phytochemical potential [67]. Similarly, research by Sikder et al. [44] highlighted the presence of flavonoid compounds in the plant’s flowers, further confirming its diverse phytochemical profile.

### 4.2. Biological Activities Calliandra surinamensis

#### 4.2.1. Membrane-Stabilizing Activity and Brine Shrimp Lethality Bioassay

The membrane-stabilizing properties of *C. surinamensis* have been explored using erythrocyte models. This activity was demonstrated by evaluating the plant’s ability to protect mice erythrocytes from hypotonic solution- and heat-induced hemolysis. According to the method outlined by Shinde et al. [68] and modified by Sikder et al. [44], the extracts exhibited significant protective effects, with concentrations as low as 2.0 mg/mL outperforming acetylsalicylic acid, a common anti-inflammatory drug. The general toxicity of *C. surinamensis* was assessed through the brine shrimp lethality bioassay using Artemia salina. This one-day in vivo investigation is an effective measure of cytotoxicity, with vincristine sulfate used as the positive control. The results of this experiment provided insight into the plant’s toxicological profile, an important consideration for its safety in medicinal applications.

#### 4.2.2. Thrombolytic and Antimicrobial Activities

The thrombolytic activity of *C. surinamensis* was evaluated based on the method developed by Prasad [69] and later modified by Silder et al. [44]. The plant extract demonstrated notable thrombolytic activity when compared to streptokinase, a standard thrombolytic agent. This indicates the potential of *C. surinamensis* in managing conditions related to clot formation, such as heart attacks and strokes. The antimicrobial properties of *C. surinamensis* were rigorously tested against thirteen bacterial strains and three fungal species. The disc diffusion method was employed to screen the plant extract, revealing broad-spectrum antimicrobial activity. The strains tested included *Bacillus cereus*, *Bacillus megaterium*, *Staphylococcus aureus*, *Escherichia coli*, *Pseudomonas aeruginosa*, and others, demonstrating the plant’s potential as a natural antimicrobial agent [70].

#### 4.2.3. Antioxidant Activity and Phytochemical Investigation

The total phenolic content of *C. surinamensis* extractives was quantified using the Folin–Ciocalteu reagent, with gallic acid as the standard. Following the methodology of Skerget et al. [71], the plant exhibited a high phenolic content, which contributes to its antioxidant activity and may play a role in its various biological effects. Phytochemical studies have revealed the presence of several bioactive compounds in the flowers of *C. surinamensis*. Notably, three flavonol glycosides were identified: 3-*O*-rhamnosylkaempferol, 3-*O*-rhamnosylmyricetin, and Myricetin-3-*O*-heptoseptanoside. The molecular structures of these compounds were elucidated using advanced spectroscopic techniques, including 1D-NMR (^1^H, ^13^C, DEPT) and 2D-NMR (COSY, HMQC, HMBC). The findings were then compared with the existing literature to validate the structures and provide further insight into the phytochemical profile of the plant [70].

## 5. Discussion

**Figure 5:**
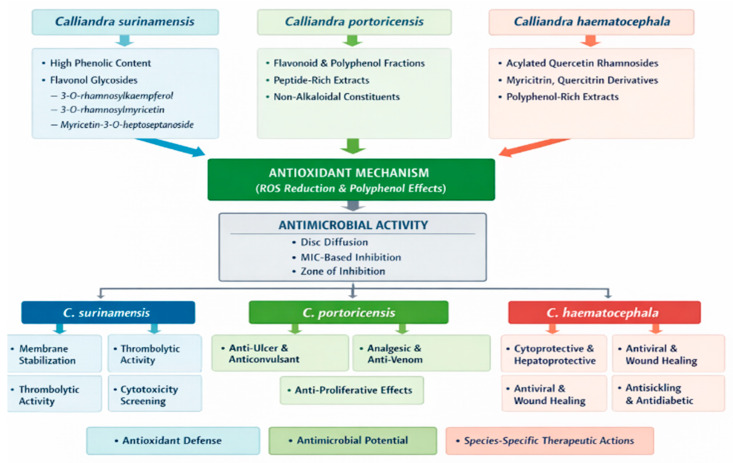
Schematic diagram showing summary of *C. portoricensis*, *C. hematocephela* and *C. surinamensis* biological activities.

The genus *Calliandra*, comprising species like *C. portoricensis*, *C. haematocephala*, and *C. surinamensis*, exhibits a rich ethnobotanical history as well as a wide range of biologically active compounds with potential pharmaceutical uses. The medicinal potential of these species in treating a variety of illnesses is revealed by this review, which integrates conventional knowledge with contemporary experimental findings. Over 60 compounds, including flavonoids, alkaloids, saponins, tannins, glycosides, and essential fatty acids, have been found through phytochemical studies conducted on various plant parts and extraction techniques. Antimicrobial, anti-ulcer, anticonvulsant, analgesic, antioxidant, anti-venom, and anti-proliferative properties are among the many biological actions that *C. portoricensis* notably demonstrates [11,13,14,16,19,20,39,58] (Table 2, Figure 5). These findings confirm its widespread application in traditional African medicine and highlight its potential for creating new therapeutic agents, especially in conditions related to oxidative stress and cancer.

Similarly, *C. haematocephala* has demonstrated notable wound-healing, antioxidant, cytotoxic, gastroprotective, antibacterial, antiviral, and hepatoprotective qualities (Figure 5). Its use in the development of nanoparticles increases its usefulness in environmental and medicinal applications. Its strong bioactivity is attributed to the presence of acylated quercetin rhamnosides and other flavonoids, indicating its significance in the fight against inflammatory conditions and microbial resistance. Biotechnological advancements have also opened a new approach of assessment of the therapeutic and environmental relevance of *C. haematocephala* used in the green synthesis of nanoparticles like zinc oxide and silver where their photocatalytic, biosensing and antimicrobial activity was determined [36]. Despite being rarely researched, *C. surinamensis* exhibits interesting pharmacological properties, including as thrombolytic, antibacterial, antioxidant, and membrane-stabilizing actions. The discovery of flavonol glycosides and other secondary metabolites validates their conventional application and creates opportunities for additional research in the treatment of viral and cardiovascular diseases.

There are still several gaps despite these encouraging findings. The translational application of *Calliandra* genera is hampered by the variation in extraction techniques, the absence of defined dosages, and the paucity of clinical evidence. Furthermore, thorough research is needed on the safety profiles, especially about pharmacokinetics and long-term toxicity. The need for thorough assessment prior to clinical application is highlighted by the dose-dependent toxicity in *C. portoricensis*. To determine efficacy and safety, future research should concentrate on identifying and defining the most powerful bioactive substances, clarifying their modes of action, and carrying out preclinical and clinical trials. Furthermore, incorporating molecular methods for precise taxonomic classification will improve pharmaceutical research’s dependability.

In addition, improvements in metabolomics and next generation GC-MS and LC-MS analysis have supported the identification and documentation of novel phyto-constituents both in *C. haematocephala* and *C. portoricensis*, contributing to drug discovery initiatives [67,72]. For taxonomic classification of *Calliandra* genus, both phylogenomic profiling and DNA barcoding have been deplored to enable precise identification of natural compounds present in the genus [2]. Computer modeling and molecular docking have made it easy to understand how *Calliandra*-based compounds could interact with their targeted site [73]. These novel advancements have not only supported ethnopharmacological prerogatives of *Calliandra* genus, but it has also been able to provide a strong approach towards translational research of viable drug candidates from *Calliandra* genera. The study of *Calliandra* genera demonstrates the potential of ethnobotanical resources in drug discovery by bringing together traditional knowledge and contemporary research. These plants could make a substantial contribution to advancing the development of sustainable, plant-based medicines that address global health issues through interdisciplinary cooperation.

## 6. Future Perspectives

The genus *Calliandra* holds immense promise for advancing natural product-based drug discovery, with species such as *C. portoricensis*, *C. haematocephala*, and *C. surinamensis* exhibiting rich ethnomedicinal histories and diverse bioactive profiles. As pharmacological research continues to evolve, further studies into the molecular mechanisms underlying their various biological activities are essential. Specifically, the exploration of *C. portoricensis*’s potential in cancer therapy, oxidative stress management, and venom-related conditions presents exciting prospects for new therapeutic agents. The plant’s promising anti-proliferative effects on cancer cells and its ability to mitigate oxidative damage and enhance endogenous antioxidant defense mechanisms suggest that it could play a significant role in managing cancer, chronic diseases, and venomous bites or stings.

Research into *C. surinamensis* offers a particularly intriguing future direction, as it demonstrated thrombolytic, antimicrobial, and antioxidant activities open the door for the development of novel natural agents for treating cardiovascular diseases, infections, and oxidative stress-related disorders. Further exploration of its bioactive compounds, such as flavonol glycosides, will contribute to the discovery of new drug candidates targeting inflammation, infections, and oxidative stress. Additionally, *C. surinamensis*’s potential as a natural thrombolytic agent could be of great interest in areas with limited access to conventional thrombolytic drugs, offering an alternative with reduced side effects.

For *C. haematocephala*, ongoing research into its antimicrobial, antiviral, and hepatoprotective properties presents an opportunity for the discovery of novel agents combating resistant pathogens, particularly in the face of growing antimicrobial resistance. Moreover, its potential role in nanoparticle synthesis for applications in biosensing, environmental pollution remediation, and drug delivery systems paves the way for the integration of plant-based nanotechnology into modern biomedical applications. Multidisciplinary research, incorporating pharmacology, nanotechnology, and biotechnology, will be essential in fully harnessing the therapeutic potential of these plants.

The safety profiles of these species, especially concerning their toxicological effects and pharmacokinetics, require further investigation to ensure their safe medicinal application. Comprehensive toxicity studies and long-term administration trials are necessary to evaluate potential adverse effects and establish safe dosages, facilitating their eventual clinical use. Also, molecular techniques such as omics and advanced metabolomics should be deployed to map the chemo-diversity of the *Calliandra* genus to identify important biosynthetic pathways and important active secondary metabolites. While some of its pharmacological (anti-inflammatory, anti-sickling, and anticancer) properties have been explored, it was discovered that most were performed in vitro. Therefore, there is a need for more in vivo mechanisms of action and pharmacological elucidation to justify observations from preliminary investigations.

## 7. General Conclusions

In conclusion, *Calliandra* genera, including *C. portoricensis*, *C. haematocephala*, and *C. surinamensis*, represent a valuable source of bioactive compounds with significant therapeutic potential. The diverse pharmacological properties demonstrated by these plants—ranging from antimicrobial, anti-inflammatory, and anti-cancer activities to hepatoprotective, thrombolytic, and antioxidant effects—underscore their role in addressing unmet medical needs in both modern and traditional medicine. However, to fully realize their medicinal value, future research must focus on isolating and characterizing key bioactive compounds, optimizing extraction methods, and conducting rigorous preclinical and clinical trials.

The pharmacological exploration of *Calliandra* genera, coupled with an understanding of their safety profiles, will be crucial in advancing their therapeutic applications. Furthermore, sustainability and ecological considerations should guide the cultivation and use of these plants, ensuring their availability for future generations. As research into the medicinal, ecological, and biotechnological applications of *Calliandra* genera expands, these plants have the potential to make significant contributions to the fields of pharmacology, nanotechnology, and environmental sustainability, ultimately leading to the development of new, plant-based therapeutic agents that benefit global health.

## Figures and Tables

**Table 1:** Specific Phytochemical compounds identified in *C. portoricensis*, *C. hematocephela* and *C. surinamensis*.

Compound Number	Compound Name	Chemical Class	Plant Part	Extraction Solvent	Identification Method	References
**Fatty Acids and Derivatives**						
C-1	Hexadecanoic acid (Palmitic acid)	Fatty Acid	Leaves, Root & Bark	Hexane	Confirmed (GC-MS vs. Standard)	[26,46]
C-2	Hexadecanoic acid, methyl ester	Fatty Acid Ester	Root bark	Methanol	Tentative (GC-MS/NIST)	[17]
C-3	Hexadecanoic acid ethyl ester	Fatty Acid Ester	Root & Bark	Hexane	Tentative (GC-MS/NIST)	[26]
C-4	Tetradecanoic acid	Fatty Acid	Root	Chloroform	Tentative (GC-MS/NIST)	[27]
C-5	Methyl tetradecanoate	Fatty Acid Ester	Root bark	Methanol	Tentative (GC-MS/NIST)	[17]
C-6	Linoleic Acid	Fatty Acid	Root, Leaves	Hexane, Ethyl acetate, Methanol	Confirmed (GC-MS vs. Standard)	[46,47]
C-7	Linolenic acid	Fatty Acid	Leaves	Hexane	Confirmed (GC-MS vs. Standard)	[46]
C-8	Oleic acid	Fatty Acid	Leaves	Hexane	Confirmed (GC-MS vs. Standard)	[46]
C-9	cis-13-Octadecenoic acid	Fatty Acid	Root bark	Methanol	Tentative (GC-MS/NIST)	[17]
C-10	Methyl 9-cis, 11-trans-octadecadienoate	Fatty Acid Ester	Root bark	Methanol	Tentative (GC-MS/NIST)	[17]
C-11	9-Hexadecenoic acid methyl ester	Fatty Acid Ester	Root & Bark	Hexane	Tentative (GC-MS/NIST)	[26]
C-12	6-Octadecenoic acid methyl ester	Fatty Acid Ester	Root	Chloroform	Tentative (GC-MS/NIST)	[27]
C-13	Z-7-Tetradecenoic acid	Fatty Acid	Root	Chloroform	Tentative (GC-MS/NIST)	[27]
C-14	14-Methylpentadecanoic acid methyl ester	Fatty Acid Ester	Root & Bark	Hexane	Tentative (GC-MS/NIST)	[26]
C-15	14-Methylhexadecanoic acid methyl ester	Fatty Acid Ester	Root & Bark	Hexane	Tentative (GC-MS/NIST)	[26]
C-16	Methyl stearate	Fatty Acid Ester	Root bark	Methanol	Tentative (GC-MS/NIST)	[17]
**Phenolic Acids**						
C-17	Gallic acid	Phenolic Acid	Root bark, Stem, Leaves	Methanol, Ethyl Acetate	Confirmed (NMR/HPLC)	[13,48]
C-18	Methyl gallate	Phenolic Acid Ester	Stem, Leaves	Ethyl Acetate	Confirmed (NMR)	[48]
C-19	p-Hydroxybenzoic acid	Phenolic Acid	Bark	Ethyl Acetate	Confirmed (NMR/HPLC)	[35]
C-20	Protocatechuic acid	Phenolic Acid	Bark	Ethyl Acetate	Confirmed (NMR/HPLC)	[35]
C-21	Caffeic acid	Phenolic Acid	Bark	Ethyl Acetate	Confirmed (NMR/HPLC)	[35,48]
C-22	Ferulic Acid	Hydroxycinnamic Acid	Root bark	Methanol	Tentative (GC-MS/NIST)	[13]
**Flavonoids & Tannins**						
C-23	Catechin	Flavan-3-ol	Twigs, Stem bark	Acetone	Confirmed (NMR)	[49]
C-24	Epicatechin	Flavan-3-ol	Twigs, Stem bark	Acetone	Confirmed (NMR)	[49]
C-25	Epigallocatechin	Flavan-3-ol	Leaves	Acetone	Confirmed (NMR)	[49]
C-26	Epigallocatechin-3-*O*-gallate	Flavan-3-ol Gallate	Leaves, Twigs, Stem bark	Acetone	Confirmed (NMR)	[49]
C-27	Afzelechin	Flavan-3-ol	Root bark	Methanol	Tentative (GC-MS/NIST)	[13]
C-28	Quercitrin (Quercetin-3-*O*-rhamnoside)	Flavonol Glycoside	Stem, Leaves, Aerial parts	Ethyl Acetate	Confirmed (NMR)	[46,48]
C-29	Myricitrin (Myricetin-3-*O*-rhamnoside)	Flavonol Glycoside	Stem, Leaves	Ethyl Acetate	Confirmed (NMR)	[48]
C-30	Isoquercitrin (Quercetin-3-*O*-glucoside)	Flavonol Glycoside	Stem, Leaves	Ethyl Acetate	Confirmed (NMR)	[48]
C-31	Afzelin (Kaempferol-3-*O*-rhamnoside)	Flavonol Glycoside	Stem, Leaves	Ethyl Acetate	Confirmed (NMR)	[48]
C-32	Astilbin	Flavanone Glycoside	Bark	Ethyl Acetate	Confirmed (NMR)	[35]
C-33	Neoisoastilbin	Flavanone Glycoside	Bark Ethyl	Acetate	Confirmed (NMR)	[35]
C-34	Catechin-3-*O*-rhamnoside	Flavan-3-ol Glycoside	Bark Ethyl	Acetate	Confirmed (NMR)	[35]
C-35	Myricetin	Flavonol	Aerial parts	Ethyl Acetate	Confirmed (NMR/HPLC)	[46]
C-36	Kaempferol	Flavonol	Aerial parts	Ethyl Acetate	Confirmed (NMR/HPLC)	[46]
C-37	Quercetin 3-*O*-methyl ether	Flavonol	Stem, Leaves	Ethyl Acetate	Confirmed (NMR)	[48]
C-38	Myricetin 3-*O*-*β*-D-glucopyranoside	Flavonol Glycoside	Stem, Leaves	Ethyl Acetate	Confirmed (NMR)	[48]
**Galloylated Flavonoids—A key feature**						
C-39	Myricitrin 2″-*O*-gallate	Galloylated Flavonol	Stem, Leaves	Ethyl Acetate	Confirmed (NMR)	[48]
C-40	Myricitrin 3″-*O*-gallate	Galloylated Flavonol	Stem, Leaves	Ethyl Acetate	Confirmed (NMR)	[48]
C-41	Myricitrin 2″,3″-*di*-*O*-gallate	Galloylated Flavonol	Stem, Leaves	Ethyl Acetate	Confirmed (NMR)	[48]
C-42	Quercitrin 2″-*O*-gallate	Galloylated Flavonol	Stem, Leaves	Ethyl Acetate	Confirmed (NMR)	[48]
C-43	Quercitrin 2″,3″-*di*-*O*-gallate	Galloylated Flavonol	Stem, Leaves	Ethyl Acetate	Confirmed (NMR)	[48]
C-44	Afzelin 2″-*O*-gallate	Galloylated Flavonol	Stem, Leaves	Ethyl Acetate	Confirmed (NMR)	[48]
C-45	Afzelin 3″-*O*-gallate	Galloylated Flavonol	Stem, Leaves	Ethyl Acetate	Confirmed (NMR)	[48]
C-46	Myricetin-3-*O*-(2″,3″-*di*-*O*-galloyl)-rhamnopyranoside	Galloylated Flavonol	Aerial parts	Ethyl Acetate	Confirmed (NMR)	[46]
C-47	Keampferol-3-*O*-(2″-*O*-galloyl)-rhamnopyranoside	Galloylated Flavonol	Aerial parts	Ethyl Acetate	Confirmed (NMR)	[46]
C-48	1,2,3,4,6-penta-*O*-galloyl-*β*-D-glucopyranose	Hydrolyzable Tannin	Stem, Leaves	Ethyl Acetate	Confirmed (NMR)	[48]
**Terpenes & Steroids**						
C-49	Lupeol	Triterpenoid	Root, Leaves	Hexane, Ethyl acetate, Methanol	Confirmed (NMR)	[46,47]
C-50	Spinasterone	Steroid	Root	Hexane, Ethyl acetate, Methanol	Confirmed (NMR)	[47]
C-51	Squalene	Triterpenoid	Root	Chloroform	Tentative (GC-MS/NIST)	[27]
**Other Compounds**						
C-52	Zapotin	Benzopyran	Root bark	Methanol	Tentative (GC-MS/NIST)	[13]
C-53	Lunularin	Dihydrostilbenoid	Root bark	Methanol	Tentative (GC-MS/NIST)	[13]
C-54	Equol	Isoflavan	Root bark	Methanol	Tentative (GC-MS/NIST)	[13]
C-55	1-Methyl-3-phenylindole	Indole Derivative	Root bark	Methanol	Tentative (GC-MS/NIST)	[17]
C-56	Hydroxymethyl anthraquinone	Anthraquinone	Root, Leaves	Crude Methanol	Tentative (Color Test/GC-MS)	[26]
**Aliphatic Hydrocarbons**						
C-57	Undecane	Hydrocarbon	Root & Bark	Hexane	Tentative (GC-MS/NIST)	[26]
C-58	Dodecane	Hydrocarbon	Root & Bark	Hexane	Tentative (GC-MS/NIST)	[26]
C-59	Tetradecane	Hydrocarbon	Root & Bark	Hexane	Tentative (GC-MS/NIST)	[26]
C-60	1-Octadecene	Hydrocarbon	Root bark	Methanol	Tentative (GC-MS/NIST)	[17]
C-61	1,8,11-Heptadecatriene	Hydrocarbon	Root	Chloroform	Tentative (GC-MS/NIST)	[27]
C-62	1,19-Eicosadiene	Hydrocarbon	Root	Chloroform	Tentative (GC-MS/NIST)	[27]

Generic classes detected only by color tests (e.g., Alkaloids, Saponins) have been removed and are discussed in the text for context. Compound numbers (e.g., C-1, C-2) correspond to the chemical structures presented in a separate, standardized figure.

**Table 2:** Summary of phytochemicals and biological activities of *C. portoricensis*, *C. haematocephala*, and *C. surinamensis*.

Calliandra Genus	Key Phytochemicals	Biological Activity
*C. portoricensis*	Fatty Acids & Esters: Hexadecanoic acid (Palmitic acid), Methyl stearate, Linoleic acid, Oleic acid, cis-13-Octadecenoic acid. Phenolic Acids: Gallic acid, Ferulic Acid. Flavonoids & Tannins: Catechin, Epicatechin, Afzelechin. Terpenes & Steroids: Squalene. Other Compounds: Zapotin, Lunularin, Equol, 1-Methyl-3-phenylindole, Hydroxymethyl anthraquinone. Aliphatic Hydrocarbons: 1-Octadecene, Undecane, Dodecane, Tetradecane	Antimicrobial & Anti-Ulcer—Active against *S. aureus*, *E. coli*, *K. pneumoniae*, *C. albicans*; gastroprotective in ulcer models. Anticonvulsant & Analgesic—Protective in pentylenetetrazole and electroshock models; reduces pain in writhing and formalin tests. Antioxidant & Anti-Venom—Mitigates oxidative stress; reverses venom-induced hepatotoxicity and hematological alterations. Anti-Proliferative & Cytotoxic—Inhibits growth of prostate (PC-3, LNCaP, DU-145) and breast (MCF-7) cancer cells; induces apoptosis.
*C. haematocephala*	Phenolic Acids: Gallic acid, Methyl gallate, p-Hydroxybenzoic acid, Protocatechuic acid, Caffeic acid. Flavonoids & Tannins: Quercitrin, Myricitrin, Isoquercitrin, Afzelin, Myricetin, Kaempferol, Catechin, Epicatechin, Epigallocatechin. Galloylated Flavonoids: Quercitrin 2″-*O*-gallate, Myricitrin 2″,3″-*di*-*O*-gallate, Afzelin 2″-*O*-gallate, 1,2,3,4,6-penta-*O*-galloyl-*β*-D-glucopyranose. Terpenes & Steroids: Lupeol, Betulinic acid.	Antioxidant & Cytoprotective— Strong radical scavenging; protects against gastric lesions. Antimicrobial & Antiviral—Antibacterial against *S. typhi*, *S. aureus*, *E. coli*; reduces rotavirus severity. Anti-inflammatory & Hepatoprotective—High anti-inflammatory potency (87.80%); protects against CCl_4_-induced liver injury. Wound Healing & Antidiabetic— Enhances wound tensile strength; inhibits α-amylase and α-glucosidase. Antisickling—High inhibitory and reversal effects on sickled red blood cells. Nanomedicine—Used in green synthesis of silver and zinc oxide nanoparticles with antibacterial properties.
*C. surinamensis*	Flavonoids & Tannins: 3-*O*-Rhamnosylkaempferol, 3-*O*-Rhamnosylmyricetin, Myricetin-3-*O*-heptoseptanoside, Astilbin, Neoisoastilbin, Catechin-3-*O*-rhamnoside.	Thrombolytic—Shows notable clot-dissolving activity. Antimicrobial—Broad-spectrum activity against Gram-positive and Gram-negative bacteria. Antioxidant—High total phenolic content contributing to free radical scavenging. Cytotoxic—Exhibits toxicity in brine shrimp lethality assay.

## Data Availability

No new data were created or analyzed in this study. Data sharing is not applicable to this article.

## Abbreviations

*C. portoricensis*—*Calliandra portoricensis*, CH—*Calliandra haematocephala*, CS—*Calliandra surinamensis*, PTZ—Pentylenetetrazole, PC—Prostate cancer, LNCaP—Lymph Node carcinoma of the prostate, DU-145—Human prostate cancer cell, VERO—Kidney cell line, GC-MS—Gas Chromatography–Mass Spectrometry.
